# Effect of hospital attributes on patient preference among outpatient attendants in Wolaita Zone, Southern Ethiopia: discrete choice experiment study

**DOI:** 10.1186/s12913-022-07874-x

**Published:** 2022-05-17

**Authors:** Tigabu Addisu Lendado, Shimelash Bitew, Fikadu Elias, Serawit Samuel, Desalegn Dawit Assele, Merid Assefa Awato

**Affiliations:** 1 Department of Epidemiology, College of Health Sciences and Medicine, Wolaita Sodo University, Wolaita, Ethiopia; 2 Department of Reproductive Health and Nutrition, College of Health Sciences and Medicine, Wolaita Sodo University, Wolaita, Ethiopia

**Keywords:** Health care service attributes, Outpatient Care, Patient preference

## Abstract

**Background:**

Patient preference has preceded the use of health care services, and it has been affected by different hospital attributes. Meanwhile, the number of patients receiving vital health intervention is particularly low in Ethiopia. Therefore, this study aimed to determine the effect of hospital attributes on patient preference for outpatients in the Wolaita area in September 2020.

**Methods:**

A discrete choice experimental study was applied to determine the effect of hospital attributes on patient preference with a sample size of 1077. The experimental survey was conducted among outpatient attendants selected through a systematic random sampling approach. Six key attributes (competence of healthcare providers; availability of medical equipment and supplies; cost of service; wait time; distance; and hospital reputation) deduced from various hospital attributes were used to elicit the patient preferences. The data was collected from participants through the Open Data Kit application. A random effect probit model with marginal willingness to pay measure and partially log-likelihood analysis was applied to extract important attributes. We used STATA version 15 software for analysis, and the fitness of the model was verified by the calculated *p*-value for the Wald chi-square with a cut-point value of 0.05.

**Result:**

One thousand forty-five patients who received outpatient care participated in the study. The random effect probit results have shown that all hospital attributes included in the study were significantly valued by patients while choosing the hospital (*p*-value < 0.001). Meanwhile, based on marginal willingness to pay and partial log-likelihood analysis, the competence of health care providers was identified as the most important attribute followed by the availability of medical equipment and supplies in hospitals.

**Conclusion and recommendation:**

The results suggested that the quality of health care providers and availability of medical equipment and supply in hospitals would be primary interventional points for improving the patient preference of hospitals. Assessment, education, and training are recommended for enhancing the quality of health care providers. And stock balance checks, inspections, and accreditation are believed to be valuable for improving the availability of equipment and supply in hospitals.

**Supplementary Information:**

The online version contains supplementary material available at 10.1186/s12913-022-07874-x.

## Introduction

The health care facility that provides service to the patient must be evaluated by integrating the patient's view, need, and values [1]. In any national or local planning effort, the accumulation of demand-side information is important within the health care system [2]. Examining the value and needs of patients and how patients prefer different attributes or characteristics of hospital care may help design health care services in the future [3].

But now a time consideration of patients' views and preferences for decision-making in the healthcare system was a major global challenge [4]. And even more in African countries [5] and even more in sub-Saharan African countries [6]. The practice of the health care system is more provider-based than patient-focused [7, 8]. On the contrary, patients look forward to participating in the decision-making process of their service or intervention [5, 9, 10]. International research indicated that patients demand more choice over their health care. They feel empowered with choice, and when provided with a choice, they exercise their right to choose [11, 12].

Patient preference over hospital is affected by organizational, environmental, and patient-related attributes. Based on different works of literature, organizational attributes that affect hospital choices are accessibility of physicians or seniors, the competence of providers, cost of services, clean, healthy environment, availability of medical supply and equipment [13–21]. In many attribute studies, distance from the facility was the primary environment attribute that prevents hospital selection [15, 16, 18–20]. And patients’ medical conditions and sociodemographic characteristics were patient-related attributes that affect the hospital's choice [13, 22, 23].

Understanding the effect of health care attributes on patient preference would help to improve patients' health care behavior [24, 25]. In turn, working according to patient preferences helps for evidence-based guidelines [26] and reduces the complexity of the ethical dilemma [27]. It has great value in improving health-related quality of life [28–33]. However, ignorance of patient preferences in hospitals decreases patient satisfaction [13] and quality of healthcare services [1]. It may also result in a waste of resources in the health care system [34].

Discrete choice experiment (DCE) is a widely used scientific method for elicitation of patient preference based on giving attributes and alternatives [35]. Hence, the DCE-based study was applied among outpatient attendants to ascertain the effect of hospital attributes on patient preference as outpatient service is the primary service area that is the first point of contact between staff and patient and the gateway for other health services [36]. It is supposed that the results of this study are essential to indicate the priority area of intervention to improve patients' preference for outpatient service.

## Methods and materials

### Study design, period, and area

Discrete Choice Experiment (DCE) design was employed to determine the effect of hospital attributes on patient preference in the Wolaita Zone from September 11 to October 13, 2020. Sodo Town is the administrative city of the Wolaita area, located 327 km far from the south of Addis Ababa, capital of Ethiopia [37]. The Wolaita Zone consists of seven hospitals, including one referral and teaching hospital, one general hospital, and five primary level hospitals. Among seven hospitals, five are public hospitals, while the two are private hospitals.

### Source and study population

All outpatients who received hospital care were our source population. And the randomly selected outpatients that received hospital services were our study population. Patients over the age of 18 took part in the study. However, those who were seriously ill or died and were referred to inpatient care were excluded.

### Sample size determination and sampling technique

The sample size was determined by R software version 3.6.3 using the step-by-step guide for sample size determination for the DCE study [38]. It was determined by the following assumptions: significance level (α = 0.05), statistical power (1-β = 80%), statistical model (random effect probit model), prior parameter estimate determined through a pilot study, and DCE design. The initial computed sample size was 979. Then, having added a non-response rate of 10%, the final sample size was determined to be 1077. The R command used to compute the sample size was described in Additional file 1.

The study was conducted in four randomly selected hospitals from seven existing hospitals in the Wolaita area. Hospitals were selected based on the type of hospital (public or private). The four hospitals picked out of seven were Dubbo St. Mary Primary Hospital, Bombe Primary Hospital, Bitena Primary Hospital, and Wolaita Sodo Referral and Teaching Hospital. The proportional allocation of the sample size for each selected hospital was done based on the monthly outpatient attendants. Finally, study participants were selected using a systematic random sampling method at every tenth interval, excluding those who were seriously ill or died and referred to inpatient care.

### Data collection procedure, instrument, and measurement

The data collection was done by six qualified public health officers who were working at the hospital. They have experience in data collection with the Smart Mobile Open Data Kit (ODK) application. The software version used to collect the data was ODK Collect v1.26.1. Four supervisors specialized in Master of Public Health were assigned to supervise. And they oversee the data collectors daily while collecting the data to ensure the data were realistic. Further, the researchers checked choice consistency (consistency of the patients’ responses) daily. At data collection time, the valuable method of preventing COVID-19 infections (respiratory hygiene, hand hygiene, and physical distance) was rigorously followed by data collectors.

A structured questionnaire was used to interview the participants. It was written in English and translated into national (Amharic) and local (Wolaitegna) languages. The questionnaire has three different versions. These different versions can help patients for looking all the attributes while choosing the hospital [12]. The questionnaire has comprised of patient characteristics and the DCE questionnaire. The questions related to patient characteristics included were sociodemographic characteristics of the patient, the type of hospital, and the frequency of hospital visits.

A DCE questionnaire consists of a common disease scenario, warmup, and main choice questions. The disease scenario was first presented during the interview, followed by a warmup and main choice questions. Disease scenario and warmup choice questions help to position the study participants in a hypothetical situation. The warmup choice question also helps the patient become familiar with the choice questionnaire, but it was not part of the final analysis [32].

The DCE choice questions were presented to patients in the form of “Table 1”. Then, the patients will prefer the options in two hypothetical hospitals, i.e. A or B that presented with different attribute levels. The time required to complete the DCE survey varied from 25 to 40 min, depending on the assessment conducted during the pilot study.

**Table 1:** Sample of the choice set presented in the choice questionnaire

Attributes	Hospital A	Hospital B
**Waiting time**	30 min	60 min
**Service cost**	0 ETB	500 ETB
**Distance from hospital**	Far	Near
**Hospital Reputation**	Moderate Reputation	Moderate Reputation
**Health care provider competence**	Moderate Competence	Poor Competence
**Availability of medical equipment and supply**	Not Available	Partially Available
**Which hospital do you prefer?**	**Hospital A**	**Hospital B**

### Steps of discrete choice experiment design

#### Establishing attributes and levels

**Fig. 1:**
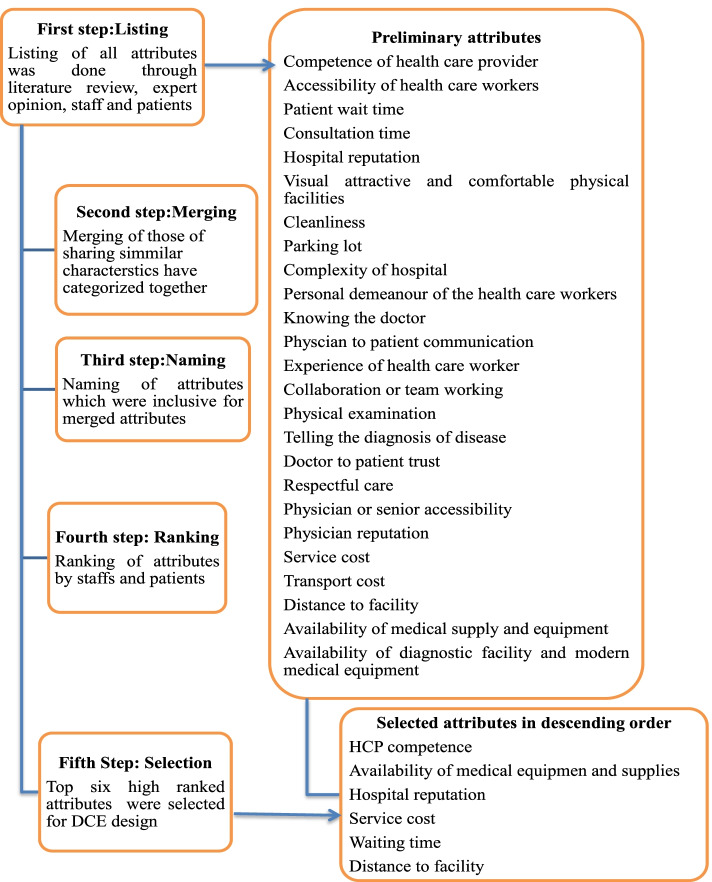
Steps of attributes selection for DCE design

Hospital attributes that can influence the patient preference of hospital identified through literature review [13, 39–42] and opinions gained from health care workers and patients. Then, the identified attributes were listed and ranked by health workers and patients to extract the most important one for DCE design. Finally, six key attributes were identified for DCE design, and the steps are outlined in  Fig. 1. Similarly, the level assigned to attribute was evaluated by health care workers and patients. The attributes and their levels used in DCE design were conceptually defined in Table 2. The patients' wait-time and service cost levels were ensured by the patients' wait-time until consulted by providers and the costs expended for given service which was estimated from 20 outpatients. In addition, the patients were asked about their willingness to wait and pay to get the service from the hospital.

**Table 2:** Attributes and levels of health care service for determining the preference of hospital toward attributes of health care service in Wolaita Zone, 2020

Attributes	Levels	Conceptual Definition
Health care provider competence	-Good competent HCP -Moderate competent HCP -Poor competent HCP	The competence of HCP was categorized based on technical skills, knowledge, ability, physician–patient communication, team care, empathy, trust, and respectful care of health care providers
Availability of medical equipment and supply	-Fully available equipment and supply -Partially available equipment and supply -Not available	The availability of equipment and drug supply was classified according to the availability of all major examination or intervention equipment or drugs, and the supply was available at a needed time in the hospital
Distance from the hospital	-Near to the hospital -Distant from hospital	The distance from the facility was classified according to the distance from the patient's resident (those residing less than 5 km from the hospital were classified as near to the hospital and otherwise distant)
Hospital Reputation (Other patients rating of hospital)	-Good hospital reputation -Moderate hospital reputation -No information on hospital reputation -Poor hospital reputation	The hospital's reputation was ranked based on information received from other patients on the overall hospital rating. This is based on their experience with the hospital, including cleanliness, the quality of the rooms, the quality of the food, the friendliness and communication skills of the staff, and the personal quality of the physician
Waiting time	-1/2 h -1 h -3 h	Wait times were classified based on time spent in the hospital until consultation with care providers and classification was based on responses from twenty respondents
Service Cost	-0 ETB -100 ETB -300 ETB -500 ETB -1000 ETB	The cost of the service was classified based on the response of 20 respondents and evaluated based on the amount the person has to pay for the service

In this way, the cost was categorized into five-level and the wait time into three-level. The lowest level was 0 ETB in service charge because three out of the twenty respondents who have insurance did not want to spend an extra charge for their outpatient service. The highest level was 1,000 ETB in service charges because four out of the twenty respondents were willing to pay as much as 1,000 ETB for their provided service. The lowest wait time level was 30 min because six out of the twenty respondents didn't feel comfortable waiting more than 30 min. And the highest wait time level was 2 h because three out of the twenty respondents will tolerate waiting for 2 h to get the service from the hospital. Intermediate service cost and wait-time levels were selected to ensure good coverage.

#### Constructing experimental design

Development of design with full factor design results on 1080 (${3}^{3} *{2}^{1} *{4}^{1} *{5}^{1}$) possible choice scenarios that put the cognitive burden on respondents. It was calculated based on ${a}^{n} x {b}^{m}$ where *a* and *b* are the different attribute levels and *n* and *m* are the different attributes. Therefore, D-optimal design was created by the “idefix" package in R software version 3.6.3 to decrease the choice scenarios with increased efficiency of parameter estimate Fourteen choice sets were created with prior information gained from the pilot study for a precise estimate of parameters. Then, the choice sets created were converted to questionnaire survey format using the "shining" application using the Surveyapp function. Each choice-set was checked for the dominant choice or utility balance to increase the obtaining of preference information. The design used for this study was displayed in Additional file 2.

### Statistical analysis

The data collected via ODK was exported in CSV (comma-separated values) format using ODK Briefcase-v1.17.4 software. Then, these data were arranged in a long-form following user-guide case study by WHO on a discrete choice experiment [12] and imported into the STATA software version 15 for analysis. When organizing the data in long-form, a pair of observations were for a choice-set. A respondent who has answered all the choice questions (14 choice-set) contributes to twenty-eight observations, and the other characteristics of the patient remain constant over it. Wait times and service costs have been coded as they appear, and other qualitative attributes have been coded by dummy coding. The dependent variable was recoded as "1" for the chosen option and "0" for the not chosen option by the respondent.

The random-effect binary probit model was used to analyze the panel-based binary response. The model has considered the variation in respondent preference by declaring the nature of the panel response before starting the regression. With regression, the variance at the panel level, or the heterogeneity of preferences among respondents was defined by the likelihood ratio test of rho *(ρ)* with a p-value less than 0.05. The rho (ρ) was a proportion of the total variance attributed to the variance component at the panel level. As shown in Table 4, the p-value was less than 0.05, indicating the heterogeneity of preferences amongst respondents.

The analysis follows the random utility theory which assumes the individual will choose the alternatives that are associated with the highest utility. Thus, individual *n* will choose alternative *i* over *j* if and only if: -

1 $${\mathbf{U}}_{\mathbf{n}\mathbf{i}}>{\mathbf{U}}_{\mathbf{n}\mathbf{j}} {\forall }_{\mathbf{i}}\ne \mathbf{j}\in$$

where *U* is the utility for a given option.

As DCE relies on the stochastic assumption, unobserved factors influencing the patient decision were considered in the study.

2 $${\mathrm{U}}_{\mathrm{in}}={\mathrm{V}}_{\mathrm{in}}+{\upvarepsilon }_{\mathrm{i}}+{\upmu }_{\mathrm{n}}$$

3 $$V_in= \beta_0+\beta_1 \Delta comp+\beta_2 \Delta equip +\beta_3 \Delta cost+\beta_4 \Delta reput+\beta_5 \Delta distance+ \beta_6 \Delta WT+\varepsilon $$

where, V_ni_ is a vector of observed variables relating to alternative *i* for person *n*, ɛ_i_ is the random error term that includes random variation across discrete choices and it follows a normal distribution, µ*n* is the random error term across respondents and is constant for individual, β_0_ is a constant coefficient, β_1-6_ were coefficient for six main attributes level and Δ indicates the difference in attribute levels.

The effect of hospital attributes on patients' preferences was evaluated primarily by patients’ values for six key attributes. The patients’ values of the attributes while choosing a hospital were assessed by looking at its coefficient (β), significance (95% CI), and direction of attributes estimate. The estimated coefficient has been interpreted with the z-score or probit index. Secondly, marginal willingness to pay (MWTP) measure and the partial log-likelihood analysis were used to prioritize or rank the effect of the attribute on the patient preference of the hospital [43–45].

MWTP was determined for the amount of money willing to pay for a particular hospital attribute. The "n*lcom*" command in STATA was used to test its statistical significance by 95% CI (Delta method). The MWTP results were interpreted as the patients' willingness to pay for service with an improvement of another attribute. And a partial log-likelihood analysis was estimated using the log-likelihood change in the omission of one variable at a time and estimating its difference. If the difference is large, that variable was considered more important than the other variables. The fitness of the model was verified by checking if there was a significant difference between the intercept model and the complete model with a calculated p-value for Wald chi-square (X2) with a cut-point value of 0.05.

Moreover, the sensitivity analysis was conducted in this study to know the average probability changes in hospital preference with average marginal effect. It was analyzed with the post-estimation command in STATA. Average marginal effect compares average probability change in hospital preference for a change in attribute levels relative to baseline levels.

### Quality assurance

The validity and quality of the discrete experimental study were checked by a checklist developed by two authors Louverie and Lancer in 2008 [32]. The theoretical validity of the study was reviewed by evaluating whether the estimated parameters matched the expected signs. A choice consistency was checked by repeating the fourth choice-set twice in the DCE questionnaire, and the calculated scale reliability coefficient was 0.95. This coefficient has ensured high consistency of patients’ responses to choice questions. But the choice set repeated was not included in the final analysis. Also, to assure the quality, a pilot study was undertaken with 50 respondents outside the study area at the Bale Primary Hospital in the Wolaita Zone. The prior parameter estimates obtained from the pilot study were used for sample size calculation, and their prior estimates were described in Additional file 3.

## Result

### Socio-demographic and patient characteristics

The total response rate to the question was 97.03%, resulting in the completion of one thousand and forty-five outpatients out of a total sample of one thousand and seventy-seven. It results in a total observation of 29,260 with two observations corresponding to a choice set. The median age of participants was 35 years, ranging from 27 to 45 years, with 49% of respondents aged 35 to 64. Of the total participants, 608 (58.02%) were urban dwellers; 586 (56.1%) were men, and 359 (34.5%) have completed a diploma or higher level of education. Of the total participants, 749 (71.7%) visited the public hospital, and 403 (38.6%) were insured. The result of the patients' characteristics of the participants included in the study was presented in “Table 3”.

**Table 3:** Sociodemographic characteristics of the respondents among outpatient attendants (*N* = 1045

Characteristics		Number	Percent
Age	18–34	494	47.3
	35–64	520	49.8
	> 64	31	2.9
Sex	Male	586	56.1
	Female	459	43.9
Marital status	Married	777	74.4
	Single	196	18.8
	Widowed	57	5.4
	Divorced/separated	15	1.4
Educational status	Do not read and write	147	14
	Read and write	324	31
	Primary education	60	5.7
	Secondary education	155	14.8
	Diploma and above	359	34.5
Occupation	Employed	315	30.1
	Merchant	250	23.9
	Farmer	264	25.3
	Daily laborer	76	7.3
	Student	91	8.7
	Housewife	49	4.7
Residence	Urban	608	58.2
	Rural	437	41.8
Payment status	Paying	642	61.4
	Insured	403	38.6
Type of hospital patient visited	Public Hospital	749	71.7
	Private Hospital	296	28.3
Frequency of visit to the hospital	First visit	440	42.1
	More than one times	605	57.9

## Patient’s value of attributes

Based on the result of random effect probit analysis (described in Table 4), patient values for all attributes when choosing the hospital, as indicated by their coefficients are significantly different from 0 with a p-value lower than 0.05. In this study, about 17.25 percent of the total variance was attributable to the variance component at the panel level. The utility direction was positive for provider competence: moderate (β = 0.27), good (β = 0.63); availability of medical supplies and equipment: partially (β = 0.13), fully (β = 0.57); hospital reputation: no information (β = 0.17), moderate (β = 0.087), good (β = 0.19); distance: near to the hospital (β = 0.057) as compared to their reference category. The wait time (β = -0.03) and service cost (β = -0.00025) had negative utility (negative direction) concerning the patient preference of the hospital. This coefficient and its signs were interpreted with the z-score. For example, the probability of choosing a hospital increased by a z-score of 0.63 for a hospital with good competent HCP compared to poorly competent HCP. As waiting time increases by one hour, the probability of choosing the hospital decreases by a z-score of 0.03.

**Table 4:** Random effect probit regression and average marginal effect for determining the valuing of main attributes of patient preference

Attributes	Random effect probit regression				Average Marginal Effect (Delta Method)			
	**Coefficient**	**SE**	**95% CI**		**dy/dx**	**SE**	**95% CI**	
Moderate HCP competence	0.27**	0.020	0.23	0.31	0.105	0.008	0.09	0.12
Good HCP competence	0.63**	0.021	0.59	0.68	0.24	0.0084	0.22	0.26
Partially available medical equipment and supply	0.13**	0.019	0.096	0.17	0.051	0.007	0.037	0.066
Fully available medical equipment and supply	0.57**	0.027	0.52	0.62	0.22	0.010	0.197	0.24
Near to the hospital	0.057**	0.017	0.024	0.09	0.022	0.0064	0.009	0.03
No information on hospital reputation	0.17**	0.02	0.14	0.23	0.066	0.0091	0.048	0.084
Moderate hospital reputation	0.087**	0.021	0.046	0.13	0.033	0.0082	0.017	0.049
Good hospital reputation	0.19**	0.026	0.14	0.24	0.072	0.01	0.053	0.091
Waiting time	-0.03*	0.0095	-0.046	-0.0086	-0.01	0.0036	-0.017	-0.0033
Service cost	-0.00025***	0.00003	-0.0003	-0.0002	-0.000095	0.000011	-0.00012	-0.000074
Constant	-0.43**	0.024	-0.47	-0.38				
Log of variance (lnsig2u)	-.1.5674		-4.1846	-0.3149				
Standard deviation (Sigma_u)	0.4567		0.1234	0.8543				
rho ($\rho )$	0.1725**		0.1499	0.2219				

Log likelihood = -19,533.95. Number of observations = 29,260. Number of groups = 1045. Wald *x*^2^(10) = 439.17. Prob > *x*^2^ = 0.0000. * Indicates *p*-value < 0.05 and ** if *p*-value < 0.001. dy/dx for factor levels is the discrete change from base level

### Marginal willingness to pay

Based on MWTP measurements, patients would be willing to pay an extra 2545 ETB [95% CI (FTE 1998–3093)] for a hospital with good competent HCP. Patients will also be willing to pay an extra 2287 ETB [95% CI (1773–2801 ETB)] for the hospital setting with fully available supply and equipment. It indicates that the patient preference for the hospital would be attributed to good competent HCP and the full availability of medical equipment and supply until the service expenditure exceeds 2545 ETB and 2287 ETB, respectively. The marginal willingness to pay was negative for wait times (-109.4 ETB). It indicates that the patient is willing to pay an extra 109 ETB [95% CI (183.5–35.2 ETB)] to avoid waiting for one hour “Table 5”.

**Table 5:** Marginal willingness to pay measure with 95% CI (Delta method)

Variable	MWTP	SE	95% CI	
			**Lower bound**	**Upper bound**
Moderate competent HCP	1094.5**	119.9	859.46	1329.64
Good competent HCP	2545.45**	279	1998.3	3092.6
Partially available equipment and supply	538.5**	85.7	370.6	706.5
Fully available equipment and supply	2286.8**	262	1773	2800.6
Near to the hospital	231.4*	70.15	93.8	368.8
No information about the reputation	694.8**	128.5	442.8	946.8
Moderate hospital reputation	350.3**	85.3	182.9	517.5
Good hospital reputation	760.9**	115.3	534.9	986.9
Waiting time	-109.4	37.8	-183.5	-35.2

Number of observations = 29,260. Number of groups = 1045. * Indicates *p*-value < 0.05 and ** indicates *p*-value < 0.001

### Partial log-likelihood analysis

The partial log-likelihood analysis indicated that a hospital with good competent HCP and fully available supplies and equipment collectively accounts for approximately 74.9% of the relative effect change in partial log-likelihood. As similar to the MWTP result, hospital reputation, distance from the hospital, and wait time contributed negligible effect on change in log-likelihood “Table 6”.

**Table 6:** Partial loglikelihood analysis for prioritizing important attributes among outpatient attendants in selected hospitals in Wolaita zone, 2020

Attribute level excluded from the analysis	Log likelihood	Partial effect change in log likelihood	Relative effect % change in loglikelihood	Order of effect
None	-19,533.95			
Good HCP competence	-19,973.1	-439.15	49.3	**1**
Fully availability of supplies and equipment	-19,762.1	-228.15	25.6	**2**
Moderate HCP competence	-19,624.7	-90.75	10.2	**3**
Cost of service	-19,571.3	-37.35	4.2	**4**
Good hospital reputation	-19,561	-27.05	3.03	**5**
No information about the reputation	-19,559.9	-25.95	2.9	**6**
Partially availability of supplies and equipment	-19,558.16	-24.21	2.72	**7**
Moderate hospital reputation	-19,542.5	-8.55	0.95	**8**
Near to the hospital	-19,539.8	-5.85	0.65	**9**
Waiting time	-19,538.05	-4.1	0.45	**10**
		-891.01	100	

Number of observations = 29,260. Number of groups = 1045

### Average marginal effect

**Fig. 2:**
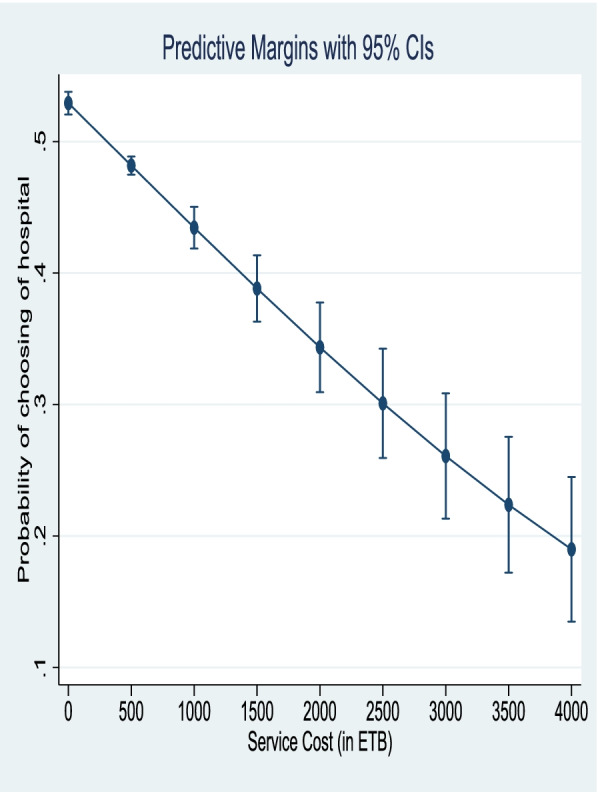
Predictive margin on choosing of the hospital among outpatient departments in selected hospitals

Based on the average marginal effect, the hospital with a moderate and good competent healthcare provider increases the probability of choice of hospital by 10.5% and 24%, respectively. On average, hospitals with partially and fully available supplies and equipment will increase the probability of the choice of hospital by 5.1% and 22%, respectively. While the probability of choosing the hospital will be decreased by 1% when the wait time for the visit by the healthcare provider increases by one hour (described in Table 4). Figure 2 shows that, on average, the probability of choosing a hospital will be decreased considerably as the service cost increases from 0 to 4,000 ETB. It will be around 52% when respondents did not pay for the service or did not pay fees and became less than 20% when patients expected to pay around 4000 ETB.

## Discussion

All attributes included in this study were significantly valued by the patient while selecting hospitals. The valuing of all attributes has validated the fact that the included attributes directly affect the patient preference of the hospital. Among the six key attributes, wait time and service cost had a negative utility. However, other attributes such as the competence of HCPs, availability of supply and equipment, the reputation of the hospital, and distance had a positive utility compared to the baseline. It indicated that the hospital setting with a low cost, less waiting time, good competent HCP, good reputation, fully available equipment, and supply are more preferred. While the hospital setting with high service cost, long wait times, poor competent HCP, poor reputation, unavailability of equipment and supply are less preferred. This sign and direction of attributes were consistent with previous DCE studies [13, 21, 46–50]. This finding indicates that policies and other decision-making issues in the health care system should be tailored to patient values.

The result has shown that the patient wants to pay a high amount of money for the hospital setting presented with good competent HCP than attribute levels. In partial log-likelihood analysis, it has contributed approximately half of the relative change in the partial effect of the log-likelihood. The hospital setting presented with moderately competent HCP was also ranked among the most important attributes and ranked third in terms of other attributes. This finding was supported by a study that has shown that the value of the patient depends more on the quality of care provided by health care workers [51]. Also, a study conducted in Canada has shown that the skills of the health care providers were the most important attributes and patients were unwilling to pay for less qualified health care providers [21]. Technical knowledge, skills, and abilities were fundamental components of the competency of health care workers. Provider competency also extends to empathy, trust, respectful care, communication, and team care [52]. Evidence shows that physician–patient communication, respectful care, and team care play an essential role in patient preference for health facilities [13, 53, 54]. This finding suggests that capacity building of health workers with extension to the varying scope of competence of health workers through various techniques such as education, training, and applying evaluation system must be considered in health care system policies.

Based on the MWTP measurement and partial log-likelihood analysis, the fully available medical equipment and supply contribute more to the patient preferences of the hospital next to good competent HCP. In this study, even the partial availability of medical equipment and supply has decreased the ranking to the seventh level. The finding of this study was consistent with the research conducted by public hospitals in the Amhara region of Ethiopia. It showed that hospitals with full drug availability and a lot of diagnostic facilities were the most important attributes for the patient preference of hospital and ranked at the top of the included attribute levels [13]. Also, another study has shown that the availability of medicine in the hospital compound has a large proportion on patient hospital choices [43, 55]. The availability and readiness of medications, devices, and technologies are critical to providing quality care [56]. This finding suggests that the minimum standard-set, stock-balance check, accreditation, and timely inspection measures might be needed to ensure the continued availability of supplies and equipment in health facilities [56].

In this study, the service cost ranked 4th in the ranking of attributes for the patient preference of the hospital. A cross-sectional study in Ethiopia identified the cost of services as a major factor influencing patient preference and access to healthcare facilities [46, 57]. The Ethiopian government recently introduced a community health insurance system as a new approach to financing health care to make the service fair and affordable.

Another finding of this study was waiting time, hospital reputation, and distance to the hospital identified as relatively less important attributes than other attributes. It was similar to another DCE study as these attributes are listed under less important attributes compared to hospital quality attributes [13, 15, 58]. The minimal strength of waiting time and distance were supported by the evidence that patients will travel further and wait longer for a higher-quality hospital [15]. Concerning hospital reputation, one study has shown that the quality of the hospital can reshape the attitude or reputation toward the hospital [59]. However, with caution, this does not mean that hospital reputation, wait time, and distance have no influence on patient preferences in the hospital.

The strength of this study was using forced-choice (Hospital A or B), which may increase information on patient preferences from estimates and reduce respondent lexicographic behavior. And the pilot study that preceded the main study was another strength of this study. The study limitation was the non-inclusion of the "opt-out option", which may cause failure to explain the behavior of some respondents. Another limitation of the study was the hypothetical nature of the choice experiment rather than the actual situation, which may cause a hypothetical bias.

## Conclusion

The patient has valued all hospital attributes included in the study while choosing the hospital. Meanwhile, the competence of the HCP and the availability of equipment and supply in the hospital were the most important attributes affecting the patient preference of the hospital. Therefore, policies and other decision-making procedures in the healthcare system should be tailored to patient priorities and needs. The researchers recommend improving the quality of healthcare providers through assessment, education, and training, as well improving the availability of equipment and supplies through stock-balance check, inspection, and accreditation.

## Supplementary Information

**Additional file 1.** Command used for sample size determination for discrete choice experiment study using R software.

**Additional file 2:** **Table 1.** DCE design for choice created by R software for eliciting patient preference for attributed related to health care services in four selected hospitals of Wolaita Zone, 2020.

**Additional file 3:** **Table 1.** Pilot study result obtained through random effect probit regression in Bale primary hospital, Wolaita, Ethiopia, 2020.

## Data Availability

The datasets used and/or analyzed as part of the ongoing study are available from the corresponding author upon reasonable request. It was not made public because the authors were preparing a new scientific paper using this data set for further processing of the publication. But, with a reasonable request, it is possible to share the dataset through the email address of the respective author.

## Abbreviations

CI: Confidence Interval
DCE: Discrete Choice Experiment
ETB: Ethiopian Birr
HCP: Health Care Provider
ODK: Open Data Kit
MWTP: Marginal Willingness to Pay
SE: Standard Error
WTP: Willingness to Pay
