# Correction: Effect of hospital attributes on patient preference among outpatient attendants in Wolaita Zone, Southern Ethiopia: discrete choice experiment study

**DOI:** 10.1186/s12913-025-13326-z

**Published:** 2025-08-13

**Authors:** Tigabu Addisu Lendado, Shimelash Bitew, Fikadu Elias, Serawit Samuel, Desalegn Dawit Assele, Merid Assefa Awato

**Affiliations:** 1 Department of Epidemiology, College of Health Sciences and Medicine, Wolaita Sodo University, Wolaita, Ethiopia; 2 Department of Reproductive Health and Nutrition, College of Health Sciences and Medicine, Wolaita Sodo University, Wolaita, Ethiopia

**Correction: BMC Health Serv Res 22, 661 (2022)**

**https://doi.org/10.1186/s12913-022-07874-x**

In this article, the author name Merid Assefa Awato was incorrectly written as Merid Asefa. The author group has been updated above and the original article has been corrected.

