# Supplementary material for: Effect of hospital attributes on patient preference among outpatient attendants in Wolaita Zone, Southern Ethiopia: discrete choice experiment study
Source: BMC Health Serv Res. 2022 May 17;22:661. doi: 10.1186/s12913-022-07874-x (PMC9110630; doi:10.1186/s12913-022-07874-x)
Supplement: Supplementary file 1 — Additional file 1. Command used for sample size determination for discrete choice experiment study using R software. [file 12913_2022_7874_MOESM1_ESM.docx]

**Command used for sample size determination for discrete choice experiment study using R software**

test_alpha=0.05

z_one_minus_alpha<-qnorm(1-test_alpha)

test_beta=0.20

z_one_minus_beta<-qnorm(1-test_beta)

parameters <- c(-1.21, 0.71, 1.07, 0.64, 1.63, 1.21, -0.93, 1.09, 1.98, -0.23, -0.37)

ncoefficients=11

nalts=2

nchoices=14

dat <- read.csv (file = file.choose(),header = T)

dat

design<-as.matrix(dat)

design

info_mat=matrix(rep(0,ncoefficients* ncoefficients), ncoefficients, ncoefficients)

expulies=exp(design%*%parameters)

for (k_set in 1:nchoices) {

alternaves=((k_set-1)*nalts+1) : (k_set*nalts)

p_set=expulies[alternaves]/sum(expulies[alternaves])

p_diag=diag(p_set)

middle_term<-p_diag-p_set%o%p_set

full_term<-(t(design[alternaves,])%*%middle_term%*%design[alternaves,])

info_mat<-info_mat+full_term

}

sigma_beta<-solve(info_mat,diag(ncoefficients))

effectsize<-parameters

N<-((z_one_minus_beta + z_one_minus_alpha)*sqrt(diag(sigma_beta))/abs(effectsize))^2
