# Supplementary material for: Effect of hospital attributes on patient preference among outpatient attendants in Wolaita Zone, Southern Ethiopia: discrete choice experiment study
Source: BMC Health Serv Res. 2022 May 17;22:661. doi: 10.1186/s12913-022-07874-x (PMC9110630; doi:10.1186/s12913-022-07874-x)
Supplement: Supplementary file 2 — Additional file 2: Table 1. DCE design for choice created by R software for eliciting patient preference for attributed related to health care services in four selected hospitals of Wolaita Zone, 2020. [file 12913_2022_7874_MOESM2_ESM.docx]

Table 1: DCE design for choice created by R software for eliciting patient preference for attributed related to health care services in four selected hospitals of Wolaita Zone, 2020

| **Scenario** | **Health Care Provider Competence** | **Availability of medical supply and equipment** | **Distance from facility** | **Hospital Reputation** | **Services Cost** | **Waiting time** |
| --- | --- | --- | --- | --- | --- | --- |
| **CS 1, Alt 1** | Poor competence | Partially Available | Far | No information | 0 | 180 |
| **CS 1, Alt 2** | Good Competence | Not Available | Close | Bad Reputation | 500 | 30 |
| **CS 2, Alt 1** | Poor competence | Partially Available | Close | Good Reputation | 100 | 180 |
| **CS 2, Alt 2** | Good Competence | Partially Available | Far | No information | 300 | 30 |
| **CS 3, Alt 1** | Poor competence | Not Available | Close | No information | 100 | 30 |
| **CS 3, Alt 2** | Poor competence | Fully Available | Close | Bad Reputation | 0 | 60 |
| **CS 4, Alt 1** | Poor competence | Partially Available | Close | Moderate Reputation | 500 | 30 |
| **CS 4, Alt 2** | Good Competence | Partially Available | Far | Bad Reputation | 100 | 60 |
| **CS 5, Alt 1** | Moderate Competence | Not Available | Close | No information | 0 | 60 |
| **CS 5, Alt 2** | Poor competence | Partially Available | Close | Bad Reputation | 300 | 30 |
| **CS 6, Alt 1** | Poor competence | Fully Available | Far | Good Reputation | 300 | 180 |
| **CS 6, Alt 2** | Good Competence | Partially Available | Far | Moderate Reputation | 0 | 180 |
| **CS 7, Alt 1** | Poor competence | Not Available | Far | Good Reputation | 0 | 30 |
| **CS 7, Alt 2** | Moderate Competence | Fully Available | Far | Moderate Reputation | 1000 | 180 |
| **CS 8, Alt 1** | Moderate Competence | Not Available | Far | Bad Reputation | 100 | 60 |
| **CS 8, Alt 2** | Good Competence | Not Available | Far | Good Reputation | 1000 | 60 |
| **CS 9, Alt 1** | Moderate Competence | Not Available | Far | Moderate Reputation | 0 | 30 |
| **CS 9, Alt 2** | Poor competence | Partially Available | Close | Moderate Reputation | 500 | 60 |
| **CS 10, Alt 1** | Moderate Competence | Not Available | Far | Moderate Reputation | 500 | 30 |
| **CS 10, Alt 2** | Moderate Competence | Fully Available | Close | No information | 500 | 180 |
| **CS 11, Alt 1** | Poor competence | Fully Available | Far | Moderate Reputation | 100 | 180 |
| **CS 11, Alt 2** | Moderate Competence | Not Available | Close | Moderate Reputation | 300 | 30 |
| **CS 12, Alt 1** | Moderate Competence | Partially Available | Close | Moderate Reputation | 1000 | 30 |
| **CS 12, Alt 2** | Good Competence | Fully Available | Close | Moderate Reputation | 0 | 180 |
| **CS 13, Alt 1** | Poor competence | Not Available | Far | Moderate Reputation | 300 | 60 |
| **CS 13, Alt 2** | Good Competence | Partially Available | Far | Bad Reputation | 500 | 180 |
| **CS 14, Alt 1** | Poor competence | Not Available | Close | Bad Reputation | 0 | 60 |
| **CS 14, Alt 2** | Moderate Competence | Partially Available | Far | Good Reputation | 500 | 60 |

**Abbreviations:** CS- Choice Set. Alt- Alternative
