# Supplementary material for: Effect of hospital attributes on patient preference among outpatient attendants in Wolaita Zone, Southern Ethiopia: discrete choice experiment study
Source: BMC Health Serv Res. 2022 May 17;22:661. doi: 10.1186/s12913-022-07874-x (PMC9110630; doi:10.1186/s12913-022-07874-x)
Supplement: Supplementary file 3 — Additional file 3: Table 1. Pilot study result obtained through random effect probit regression in Bale primary hospital, Wolaita, Ethiopia, 2020. [file 12913_2022_7874_MOESM3_ESM.docx]

## **Pilot study result**

Table 1. Pilot study result obtained through random effect probit regression in Bale primary hospital, Wolaita, Ethiopia, 2020

| **Attributes** | **Random effect probit regression** | | |
| --- | --- | --- | --- |
|  | **Coefficient (β)** | **95% CI** | |
| Moderate competence | 0.71 | 0.27 | 1.15 |
| Good competence | 1.07 | 0.51 | 1.64 |
| Close proximity to hospital | 1.21 | 0.77 | 1.65 |
| Partially available | 0.64 | 0.23 | 1.04 |
| Fully available | 1.64 | 1.17 | 2.09 |
| Poor reputation | -0.93 | -1.11 | -0.15 |
| Moderate reputation | 1.08 | 0.58 | 1.59 |
| Good reputation | 1.98 | 1.31 | 2.65 |
| Waiting time | -0.37 | -0.61 | -0.13 |
| Service cost | -0.23 | -0.32 | -0.13 |
| Constant | -1.21 | -1.98 | -0.44 |
